# A Rare Case of Mononucleosis-Induced Splenic Infarction in a Patient With Sickle Cell Trait

**DOI:** 10.7759/cureus.113178

**Published:** 2026-07-22

**Authors:** Cara Satoskar, Erin Onken, Al-Motasumbellah Al-Tamimy, Edward Griffin

**Affiliations:** 1 School of Medicine, Edward Via College of Osteopathic Medicine, Blacksburg, USA; 2 Internal Medicine, LewisGale Medical Center, Salem, USA

**Keywords:** epstein-barr virus, hematology, infective mononucleosis, sickle cell trait, splenic infarction

## Abstract

Splenic infarction is a rare complication of Epstein-Barr virus (EBV) mononucleosis, particularly in patients with underlying hematologic conditions. We present a rare case of EBV-associated splenic infarction in a 20-year-old male with sickle cell trait (SCT), highlighting the potential overlap between SCT and infection-related ischemic complications. The patient presented to the emergency department with one week of left upper quadrant pain, fever, night sweats, dyspnea, and concentrated urine following suspected dehydration after outdoor activity. Initial Monospot testing was negative. CT imaging revealed multiple splenic infarcts, and the patient was admitted with presumed splenic infarction secondary to an acute sickling crisis. His clinical course improved with supportive care, and he was discharged the following day. Peripheral blood smear obtained after discharge revealed atypical lymphocytes, and follow-up serologic testing two weeks later confirmed markedly elevated EBV viral capsid antigen IgM, supporting the diagnosis of infectious mononucleosis-associated splenic infarction. The patient was counseled on contact precautions and activity restrictions, with complete symptom resolution. Although the initial presentation suggested SCT-related vaso-occlusion, EBV was ultimately identified as the likely trigger. This case highlights the limitations of early Monospot testing and the importance of considering EBV infection in young patients with splenic infarction, even when SCT offers an alternative explanation. Additionally, it raises the possibility of overlap between SCT and infection-related ischemic complications.

## Introduction

Infectious mononucleosis (IM) caused by Epstein-Barr virus (EBV) is a common clinical syndrome predominantly affecting adolescents and young adults, classically presenting with fever, pharyngitis, and lymphadenopathy [1]. While EBV mononucleosis frequently involves the spleen, leading to splenomegaly in over 50% of cases, splenic complications such as rupture and infarction remain rare but potentially life-threatening sequelae [2,3]. Splenic rupture occurs in approximately 0.5-1% of IM cases, while splenic infarction is even less common, with its true incidence unknown [4,5]. A systematic review identified only 29 documented cases of splenic infarction associated with EBV mononucleosis in the literature between 1970 and 2023, with 70% occurring in males and approximately 80% presenting within three weeks of symptom onset [3].

The pathophysiology of splenic infarction in IM is not entirely understood but is likely multifactorial. EBV-driven lymphoid hyperplasia and infiltration of the splenic parenchyma by activated T cells responding to infected B cells leads to capsular distension and splenic fragility [2,6]. Additionally, EBV infection can induce transient prothrombotic states, including production of antiphospholipid antibodies and lupus anticoagulant, which may contribute to intrasplenic thrombosis and subsequent infarction [7,8]. Notably, 21% of reported splenic infarction cases in IM occurred in patients with underlying hematological conditions, suggesting that pre-existing coagulopathies or hemoglobinopathies may predispose to this complication [3].

Sickle cell trait (SCT), affecting approximately 300 million people worldwide with prevalence rates of 30-40% in sub-Saharan Africa and 8-10% among African Americans, is generally considered a benign carrier state [9]. However, SCT is associated with rare but well-documented complications, including splenic infarction, particularly under conditions of hypoxia, dehydration, or extreme physical exertion [9,10]. The spleen is uniquely vulnerable to sickling-related injury due to its hypoxic microenvironment and role in trapping abnormal erythrocytes [11]. While most reported cases of SCT-related splenic infarction occur at high altitude (typically above 5,000 feet), where reduced atmospheric oxygen tension promotes hemoglobin S polymerization and red blood cell sickling, cases have also been documented at sea level without apparent hypoxic triggers [3,12]. The proposed mechanism involves intrasplenic vascular occlusion by transiently sickled erythrocytes, with subsequent venous thrombosis and parenchymal ischemia [2].

The convergence of EBV-induced splenic hyperplasia and prothrombotic changes with the underlying sickling tendency of SCT raises the possibility of overlapping mechanisms contributing to splenic ischemia. This overlap highlights the potential role of infectious triggers in precipitating ischemic complications in patients with SCT and underscores the need for further investigation into the interaction between hemoglobinopathies and acute viral infections. We present a case of splenic infarction in a patient with SCT and acute EBV infection, emphasizing the importance of recognizing infectious contributors to splenic ischemia in this population.

## Case presentation

Initial hospital course

A 20-year-old male presented to the emergency department (ED) with a one-week history of progressive left upper quadrant (LUQ) abdominal pain. The pain was associated with myalgias, chills, night sweats, dyspnea, and concentrated urine. The patient reported that his symptoms began after spending an entire day outdoors with limited fluid intake, during which he felt dizzy and lightheaded. He did not take any medications, and his history was notable only for self-reported personal and family history of SCT. He denied recent travel, known sick contacts, and prior similar episodes.

On presentation, vital signs demonstrated a fever of 101.2°F, blood pressure of 164/104 mmHg, heart rate of 113 beats per minute, respiratory rate of 20 breaths per minute, and oxygen saturation of 97% on room air. Physical examination revealed tachypnea, moist mucous membranes, and tenderness to palpation in the LUQ without palpable lymphadenopathy.

Table 1 demonstrates initial laboratory studies. Labs were significant for mild leukocytosis, transaminitis, elevated lactate dehydrogenase, and an elevated D-dimer. Respiratory viral testing and a heterophile antibody (Monospot) test were performed upon initial presentation to the ED and returned negative.

**Table 1:** Laboratory investigations at initial presentation. EBV: Epstein-Barr virus; UA: urinalysis

Parameter	Patient value	Reference range
Hemoglobin (g/dL)	14.2	13.5–17.5
Hematocrit	43%	41–50%
White blood cell count (×10⁹/L)	11.8	4.5–11.0
Aspartate aminotransferase (U/L)	177	10–40
Alanine aminotransferase (U/L)	383	7–56
Alkaline phosphatase (U/L)	168	44–147
Total bilirubin (mg/dL)	1.3	0.1–1.2
Lactate (mmol/L)	0.9	0.5–2.2
Lactate dehydrogenase (U/L)	620	140–280
D-dimer (µg/mL)	2.48	<0.50
Urobilinogen (UA dipstick)	2+	Negative or trace
Specific gravity	1.020	1.005–1.030
Monospot (heterophile antibody)	Negative	Negative
COVID-19	Negative	Negative
Influenza	Negative	Negative

**Figure 1:**
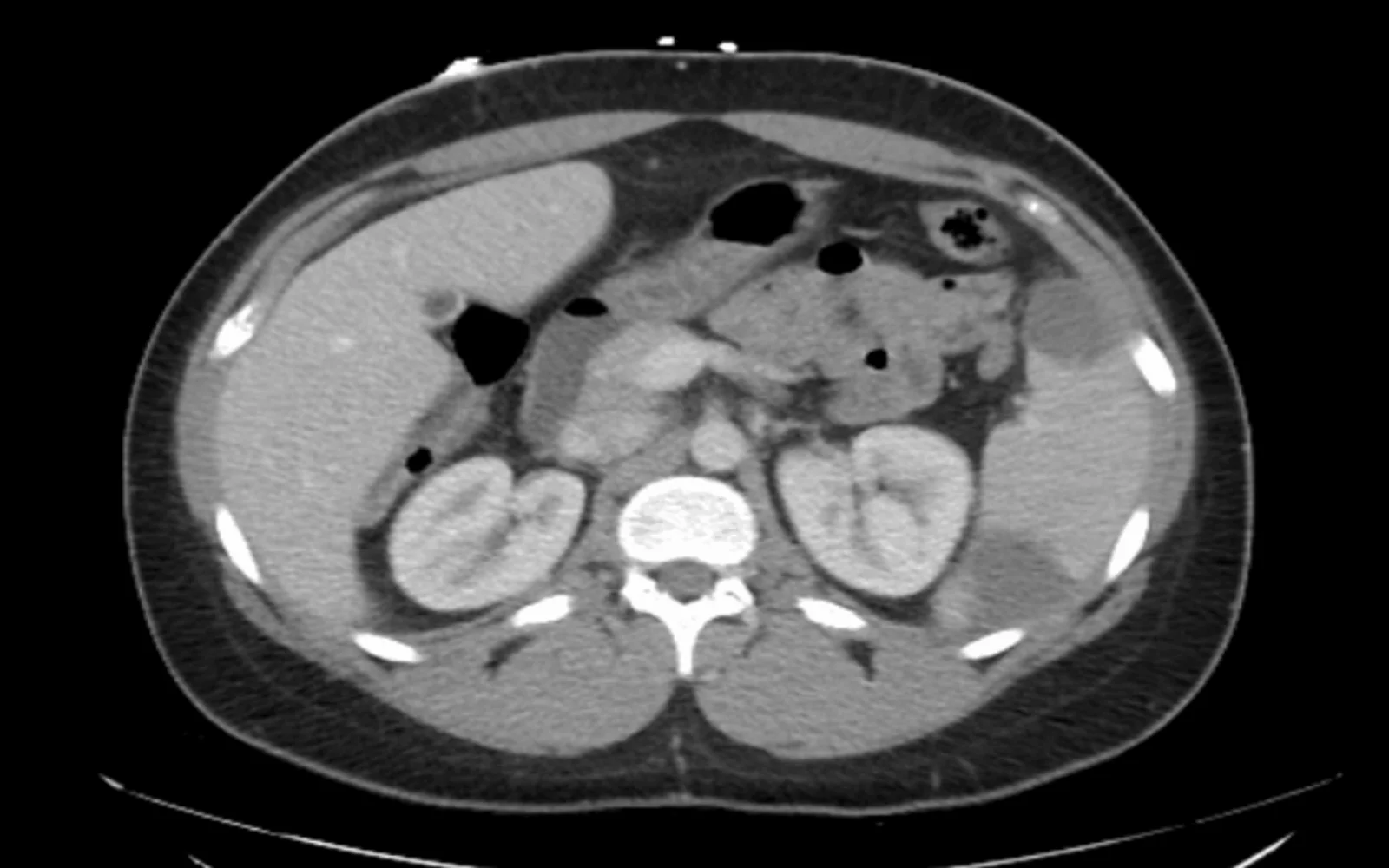
Contrast-enhanced computed tomography of the abdomen demonstrating splenomegaly with multiple peripheral wedge-shaped hypodensities consistent with splenic infarcts.

Figure 1 demonstrates computed tomography (CT) of the abdomen and pelvis, which revealed splenomegaly with multiple peripheral splenic infarcts. Given the patient’s known SCT and corresponding imaging findings, the initial working diagnosis was splenic infarction secondary to sickling. The patient was admitted for supportive care and treated with intravenous fluids and analgesics.

The patient’s abdominal pain improved during hospitalization, and he was discharged the following day with instructions to follow up with internal medicine. Due to the benign clinical course and brief admission period, hematology was not consulted during hospitalization, and further hematologic workup was deferred.

Outpatient follow-up visit

Two weeks later, he presented for outpatient follow-up and reported complete resolution of LUQ pain. Vital signs were within normal limits, and neither abdominal tenderness nor splenomegaly were appreciated on physical examination.

During this visit, additional history revealed that symptom onset followed a full day of outdoor activity at an elevation of approximately 600 feet, well below altitudes typically associated with sickling-related splenic infarction. He denied known sick contacts but reported occupational exposure to children. The absence of significant hypoxia, lactic acidosis, or severe dehydration suggested that the clinical picture was not fully consistent with a sickling-mediated event.

The peripheral blood smear report was performed during hospitalization but did not become available until after discharge. The report was reviewed at the outpatient visit and revealed normal red blood cell morphology with multiple reactive Downey type II lymphocytes. These findings prompted reevaluation for EBV infection despite the initial negative Monospot test. Subsequent EBV serologic testing revealed a markedly elevated viral capsid antigen (VCA) IgM level greater than 160 U/mL, with negative early antigen IgG, supporting acute primary EBV infection (Table 2). The patient was counseled on isolation precautions and advised to avoid contact sports due to the risk of splenic rupture.

**Table 2:** EBV-specific serologies at the two-week follow-up visit. VCA: viral capsid antigen

Parameter	Patient value	Reference range
EBV VCA IgM (U/mL)	>160	<20 (negative)
EBV early antigen IgG	Negative	Negative

## Discussion

**Figure 2:**
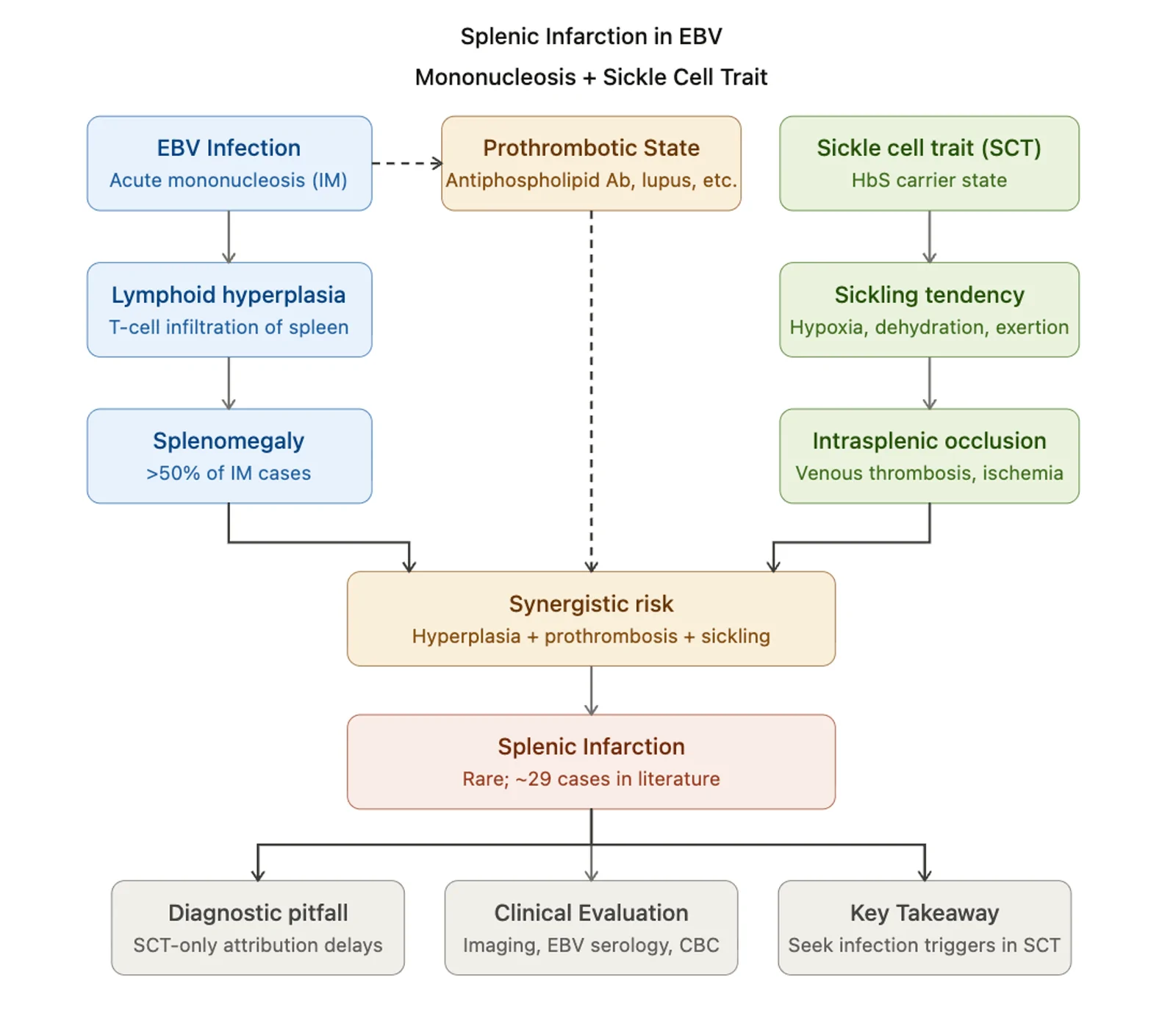
Proposed pathophysiologic mechanisms of splenic infarction in Epstein-Barr virus (EBV) mononucleosis with concurrent sickle cell trait (SCT), illustrating the potential synergistic interaction between EBV-driven splenic changes and SCT-related sickling. Original figure created by the authors in Canva.

This case highlights a rare presentation of splenic infarction associated with EBV IM in a patient with SCT. Splenic infarction remains an exceedingly rare complication of EBV mononucleosis, with only 29 reported cases between 1970 and 2023. Among these, six occurred in patients with underlying hematologic disorders, including only two reported in individuals with SCT [3]. The coexistence of EBV infection and SCT in this patient raises the possibility that overlapping infectious and hemoglobinopathy-related mechanisms may have synergistically contributed to splenic ischemia. Figure 2 illustrates the potential synergistic interaction between EBV-driven splenic changes and SCT-related sickling. This case is limited by the absence of confirmatory hemoglobin electrophoresis to quantify the hemoglobin S fraction, antiphospholipid antibody/lupus anticoagulant testing, and dedicated hemolysis markers beyond the complete blood count (CBC), none of which were pursued given the patient’s brief, benign clinical course. As such, these findings cannot establish a causal or definitively synergistic relationship between EBV infection and SCT, and the proposed interaction remains hypothesis-generating.

This case also illustrates the diagnostic challenges that arise when known risk factors are present. The patient’s history of SCT created an early, plausible explanation that may have reduced consideration of acute EBV despite the presence of classic clinical characteristics such as fever and LUQ pain in a young patient. Notably, the CBC did not indicate significant hemolysis, and the peripheral blood smear obtained during admission showed no evidence of sickling, findings that in retrospect argued against an active sickling process as the primary driver of splenic infarction. While cognitive biases such as anchoring bias can influence clinical reasoning, this case underscores the importance of reassessing initial assumptions when clinical findings are not fully consistent with expected physiologic triggers. Recognition of infectious triggers as potential contributors to splenic infarction in patients with SCT is particularly important, as attribution of infarction solely to SCT may delay identification of concurrent infectious etiologies.

Additionally, this case highlights the limitations of heterophile antibody testing in the early diagnosis of EBV infection. The sensitivity of the Monospot test varies considerably based on timing, with reported sensitivity ranging from approximately 70% to 87% and a rate of ~25% false negatives in the first week of infection [13-15]. When clinical suspicion for EBV infection remains high despite a negative heterophile test, confirmatory EBV-specific serologies, including VCA IgM and IgG, should be obtained, as these tests demonstrate sensitivities approaching 97%. VCA IgM, in particular, has greater diagnostic accuracy than heterophile antibody testing during an early infection period [15].

## Conclusions

Splenic infarction in young patients warrants a broad differential diagnosis, even in the presence of known risk factors such as SCT. This case demonstrates a rare occurrence of EBV-associated splenic infarction in a patient with SCT and highlights the potential role of infectious triggers in precipitating ischemic complications in this population. Clinicians should consider acute EBV infection in patients presenting with constitutional symptoms, transaminitis, splenomegaly, and atypical lymphocytosis, regardless of initial heterophile antibody results. Increased awareness of the potential interaction between hemoglobinopathies and infectious processes may improve diagnostic accuracy and support further investigation into the mechanisms underlying splenic ischemia in patients with SCT.
